# The respective roles of TMPRSS2 and cathepsins for SARS-CoV-2 infection in human respiratory organoids

**DOI:** 10.1128/jvi.01853-24

**Published:** 2024-11-27

**Authors:** Masatoshi Kakizaki, Rina Hashimoto, Noriyo Nagata, Takuya Yamamoto, Takashi Okura, Hiroshi Katoh, Yuki Kitai, Yukiko Akahori, Kazuya Shirato, Akihide Ryo, Kazuo Takayama, Makoto Takeda

**Affiliations:** 1 Department of Virology III, National Institute of Infectious Diseases 13511 , Tokyo, Japan; 2 Center for iPS Cell Research and Application (CiRA), Kyoto University 130382 , Kyoto, Japan; 3 Department of Pathology, National Institute of Infectious Diseases 13511 , Tokyo, Japan; 4 Medical-risk Avoidance based on iPS Cells Team, RIKEN Center for Advanced Intelligence Project (AIP), Kyoto, Japan; 5 Institute for the Advanced Study of Human Biology (WPI-ASHBi), Kyoto University 593546 , Kyoto, Japan; 6 Department of Microbiology, Graduate School of Medicine and Faculty of Medicine, The University of Tokyo 317992 , Tokyo, Japan; 7 Pandemic Preparedness, Infection and Advanced Research Center, The University of Tokyo 13143 , Tokyo, Japan; Loyola University Chicago - Health Sciences Campus, Maywood, Illinois, USA

**Keywords:** SARS-CoV-2, TMPRSS2, cathepsin, cleavage

## Abstract

A critical aspect of the mechanism of severe acute respiratory syndrome coronavirus 2 (SARS-CoV-2) infection is the protease-mediated activation of the viral spike (S) protein. The type II transmembrane serine protease TMPRSS2 is crucial for SARS-CoV-2 infection in lung epithelial Calu-3 cells and murine airways. However, the importance of TMPRSS2 needs to be re-examined because the ability to utilize TMPRSS2 is significantly reduced in the Omicron variants that spread globally. For this purpose, replication profiles of SARS-CoV-2 were analyzed in human respiratory organoids. All tested viruses, including Omicron variants, replicated efficiently in these organoids. Notably, all SARS-CoV-2 strains retained replication ability in TMPRSS2-gene knockout (KO) respiratory organoids, suggesting that TMPRSS2 is not essential for SARS-CoV-2 infection in human respiratory tissues. However, TMPRSS2-gene knockout significantly reduces the inhibitory effect of nafamostat, indicating the advantage of TMPRSS2-utilizing ability for the SARS-CoV-2 infection in these organoids. Interestingly, Omicron variants regained the TMPRSS2-utilizing ability in recent subvariants. The basal infectivity would be supported mainly by cathepsins because the cathepsin inhibitor, EST, showed a significant inhibitory effect on infection with any SARS-CoV-2 strains, mainly when used with nafamostat. A supplementary contribution of other serine proteases was also suggested because the infection of the Delta variant was still inhibited partially by nafamostat in TMPRSS2 KO organoids. Thus, various proteases, including TMPRSS2, other serine proteases, and cathepsins, co-operatively contribute to SARS-CoV-2 infection significantly in the respiratory organoids. Thus, SARS-CoV-2 infection in the human respiratory tissues would be more complex than observed in cell lines or mice.

**IMPORTANCE:**

We explored how the severe acute respiratory syndrome coronavirus 2 (SARS-CoV-2) virus infects human respiratory organoids, which are a cultured cell model made to mimic the physiological conditions of the human airways. We focused on understanding the role of different proteases of host cells in activating the virus spike proteins. Specifically, we looked at TMPRSS2, a transmembrane serine protease, and cathepsin L, a lysosomal enzyme, which helps the virus enter cells by cutting the viral spike protein. We discovered that while TMPRSS2 is crucial for the virus in certain cells and animal models, other proteases, including cathepsins and various serine proteases, also play significant roles in the SARS-CoV-2 infection of human respiratory organoids. We suggest that SARS-CoV-2 uses a more complex mechanism involving multiple proteases to infect human airways, differing from what we see in conventional cell lines or animal models. This complexity might help explain how different variants can spread and infect people effectively.

## INTRODUCTION

At the end of 2019, a severe pneumonia outbreak broke out in Wuhan, China, and spread worldwide in months (1, 2). The World Health Organization (WHO) declared a global pandemic on 11 March 2020. This unprecedented pandemic was caused by the severe acute respiratory syndrome coronavirus 2 (SARS-CoV-2) in the *Sarbecovirus* subgenus of the *Betacoronavirus* genus. The spike (S) glycoprotein of SARS-CoV-2 is an essential determinant of the virus infection mode. The S protein consists of S1, which contains a receptor-binding domain, and S2, which mediates membrane fusion (3, 4). To induce membrane fusion, cleavage of the S protein at S1/S2 and S2′ sites is necessary. S1/S2 cleavage is carried out by furin, a proprotein convertase, during S protein biosynthesis and facilitates S protein binding to the receptor ACE2 and subsequent S2′ cleavage (5, 6). Cleavage at the S2′ site occurs after binding to ACE2 (3, 5), and TMPRSS2, a type II transmembrane serine protease (TTSP) expressed on the plasma membrane, is a significant protease that mediates S2′ cleavage (3, 7, 8). Meanwhile, SARS-CoV-2 also enters the cell via endocytosis and uses cathepsin L in endosomes or lysosomes to cleave S proteins, which can also support viral entry (9).

Our research group reported that SARS-CoV-2 is efficiently isolated and cultivated in VeroE6 cells expressing TMPRSS2 (VeroE6/TMPRSS2) (7). These results build on many earlier findings that TMPRSS2 facilitates the infection of many respiratory viruses (10–14). However, these results do not indicate that TMPRSS2 is the critical protease that promotes infection of these respiratory viruses *in vivo*. We and other research groups have, therefore, demonstrated that TMPRSS2 plays a significant role in the airway replication of several respiratory viruses, including SARS-CoV-2, using mice lacking the TMPRSS2 gene (15–19).

Subsequently, numerous studies have been published on the basis that TMPRSS2 is the essential protease responsible for the cleavage activation of the SARS-CoV-2 S protein. Indeed, the emergence of the D614G mutant, followed by Alpha to Delta variants with increased TMPRSS2-using ability, is well in line with the idea that TMPRSS2 plays an essential role in the infectivity and transmissibility of SARS-CoV-2 in humans (3). However, the finding that the Omicron variant, which subsequently became the dominant strain of the pandemic, has a low capacity to utilize TMPRSS2 (20–22) raises questions about the importance of TMPRSS2 in SARS-CoV-2 infection in humans. Thus, the role of TMPRSS2 in SARS-CoV-2 infection still needs careful consideration.

Experiments using mice and cultured cells have significantly contributed to our understanding of the replication mechanisms and pathogenesis of SARS-CoV-2. However, these models may only partially reflect the virus growth in the human airways. This study analyzed various SARS-CoV-2 strains, focusing on their protease specificity using well-studied cultured cell lines and TMPRSS2 gene-knockout or gene-unmodified human respiratory organoids.

## RESULTS

### Restoration of TMPRSS2 utilization and membrane fusion capacity in Omicron subvariants

**Fig 1:**
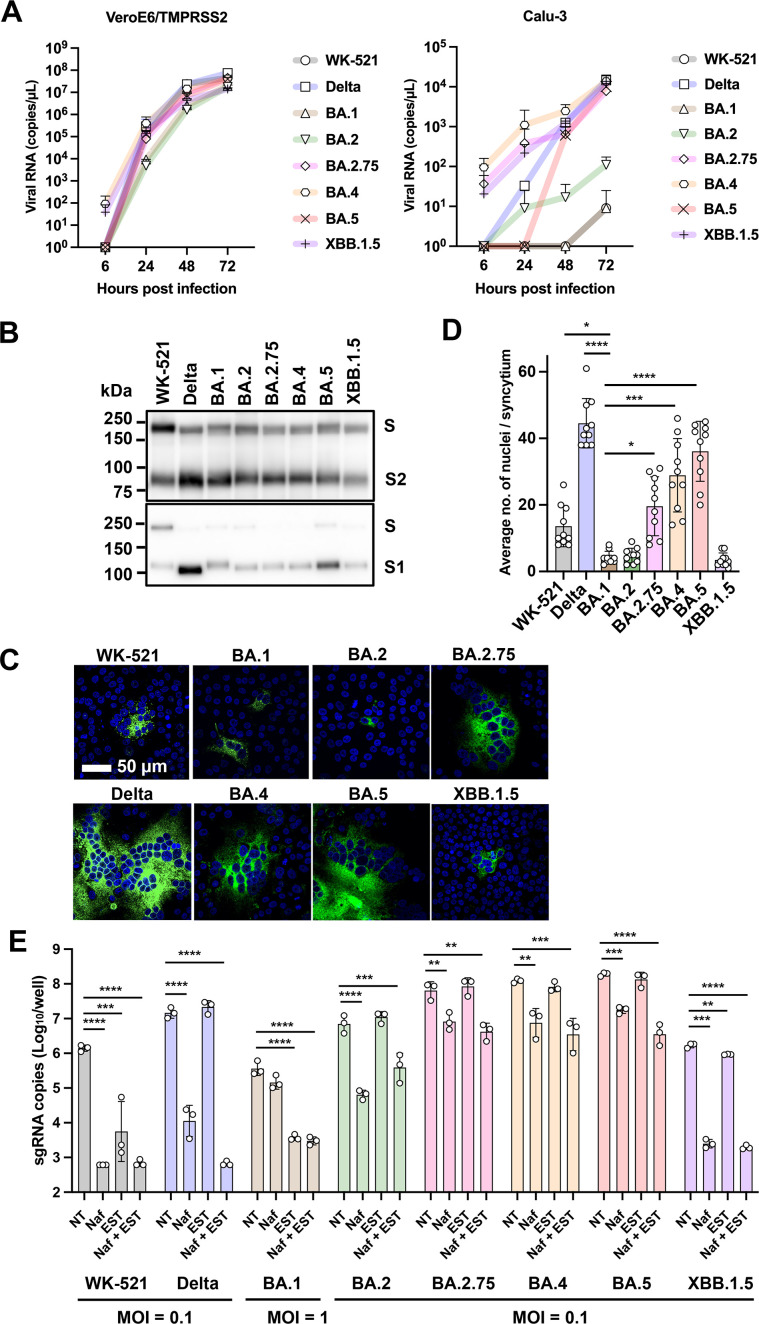
Infection in VeroE6/TMPRSS2 and Calu-3 cells. (**A**) Replication kinetics in cells infected with viruses at a multiplicity of infection (MOI) of 0.1. Viral RNA copy numbers in the culture supernatants were quantified at 6, 24, 48, and 72 h post-infection (p.i.). Error bars indicate the standard deviations of three biological replicates. Three confirmatory experiments were conducted. Mean values with positive standard deviations are shown. (**B**) S proteins detected in the culture supernatant of infected VeroE6/TMPRSS2 cells. SDS-PAGE and western blot analysis. Three confirmatory experiments were conducted. (**A, B**) (**C**) Syncytium formation in cells transfected with the S protein expression plasmid. S protein was detected by an indirect immunofluorescent assay (green) and nuclei were detected by DAPI (blue) staining. Three confirmatory experiments were conducted. (**D**) The number of nuclei in 10 individual, randomly selected syncytia shown in C was counted. Mean values ± standard deviations are shown, and significant differences were determined with one-way ANOVA. Multiple comparisons between the BA.1 spike and other variants spike were adjusted with Dunn’s multiple comparison test, *P < 0.05, **P < 0.01, ***P < 0.001, ****P < 0.0001. (**E**) Cells were infected with viruses at an MOI of 0.1 or 1.0 in the absence (NT) or the presence of either nafamostat (Naf) or EST or both (Naf +EST). At 24 h p.i., viral RNAs in cells were quantified by real-time RT-PCR. Error bars indicate the standard deviations of three biological replicates. Three confirmatory experiments were conducted. Statistical significance was determined with two-way ANOVA. Multiple comparisons between NT and different groups were adjusted with Dunnett’s multiple comparison tests, *P < 0.05, **P < 0.01, ***P < 0.001, ****P < 0.0001.

Our previous study (17) showed the replication capacities and infection modes of the original SARS-CoV-2 strain emerged in Wuhan city (WK521 strain (7)) and various variants (Alpha, Beta, Gamma, Delta, Kappa, and Omicron BA.1) in well-studied cell lines (VeroE6/TMPRSS2 and Calu-3 cells) and mice. In this study, the replication potential of Omicron subvariants was analyzed using VeroE6/TMPRSS2 cells and Calu-3 cells. VeroE6/TMPRSS2 cells express a high level of cathepsin L and TMPRSS2 (7, 23, 24). On the other hand, Calu-3 cells, a lung epithelial cell line derived from lung cancer, have a low level of cathepsins and a high level of TMPRSS2 (23). Previous studies (25, 26) have shown that SARS-CoV-2 infection in Calu-3 is dependent on TMPRSS2. As previously reported (17), in VeroE6/TMPRSS2 cells, Omicron BA.1, which has a low capacity to utilize TMPRSS2, showed similar replication potential to the TMPRSS2-utilizing Delta variant, likely because it utilizes cathepsin L efficiently in these cells. BA.1 and later subvariants (BA.2, BA.2.75, BA.4, BA.5, and XBB.1.5) also showed a high replication potential in VeroE6/TMPRSS2 cells (Fig. 1A). By contrast, in Calu-3 cells, the replication capacity of BA.1 and the WK-521 strain (7) was severely restricted, as previously reported (17). WK521 is an early epidemic strain of SARS-CoV-2 before it acquired the D681G mutation and is known to have not yet developed a high capacity to utilize TMPRSS2 (3). The Omicron subvariants after BA.1 showed an improved replication capacity in Calu-3 cells, several 10-fold recovery in BA.2, and similar levels of replication capacity to the Delta variants in BA.2.75, BA.4, BA.5, and XBB.1.5 (Fig. 1A). These results suggested that TMPRSS2-utilizing ability was restored in the Omicron subvariants. Some studies showed that an impaired S1/S2 cleavage is a characteristic of Omicron variants (20, 21, 27, 28), and they suggested that this is the main reason for the reduced membrane fusion activity of Omicron variants. However, our analysis did not confirm such a reduction in the S protein cleavage (17) (Fig. 1B). Still, the cleavage increased in all Omicron variants compared to the WK521 strain (Fig. 1B). The membrane fusion activity of the S protein of various SARS-CoV-2 strains was analyzed using a transient expression system using expression plasmids. In spite of the efficient S protein cleavage, BA.1, BA.2, and XBB.1.5 showed a marked decrease in membrane fusion activity, similar to many studies reported previously (21, 27, 28) (Fig. 1C and D). Although such a reduction in membrane fusion activity has been considered characteristic of Omicron variants, the membrane fusion activity recovered markedly in the Omicron subvariants, BA.2.75, BA.4, and BA.5 (Fig. 1C and D). These results also suggested the recovery of TMPRSS2-utilizing ability by BA.2.75, BA.4, BA.5, and XBB.1.5.

The type of protease utilized by these Omicron subvariants in Calu-3 cells was analyzed using nafamostat and EST to gain further evidence for the utilization of TMPRSS2 by recently emerged Omicron subvariants. Calu-3 cells express a low level of cathepsins (23) (Fig. S1), and thus SARS-CoV-2 infection in Calu-3 is mainly dependent on TMPRSS2 (25, 26). As reported previously (17), the infection of BA.1 was inefficient in Calu-3 cells, and a high amount of virus (multiplicity of infection [MOI] = 1.0) was used for infection. As evidenced in the previous study (17), nafamostat was unable to inhibit the infection of BA.1 in Calu-3 cells, while EST was able to inhibit it by ~2 log_10_ (Fig. 1E). These findings reconfirm that the infection of BA.1 in Calu-3 is cathepsin-dependent, despite the low expression level of cathepsins in these cells (23). On the other hand, the infection with Omicron subvariants was efficient, and a low amount of virus (MOI = 0.1) was used for infection. The infection was suppressed by nafamostat by 1–3 log_10_ but not by EST (Fig. 1E). These findings suggest a notable change in the infection mode of the Omicron variants, which were originally dependent on cathepsins but have regained the ability to utilize TMPRSS2. Furthermore, some Omicron subvariants (BA.2.75, BA.4, and BA.5) retained a significant level of infectivity even when both nafamostat and EST were added simultaneously. These results suggest the possible involvement of proteases other than serine proteases and cathepsins.

### SARS-CoV-2 infection in human respiratory organoids

**Fig 2:**
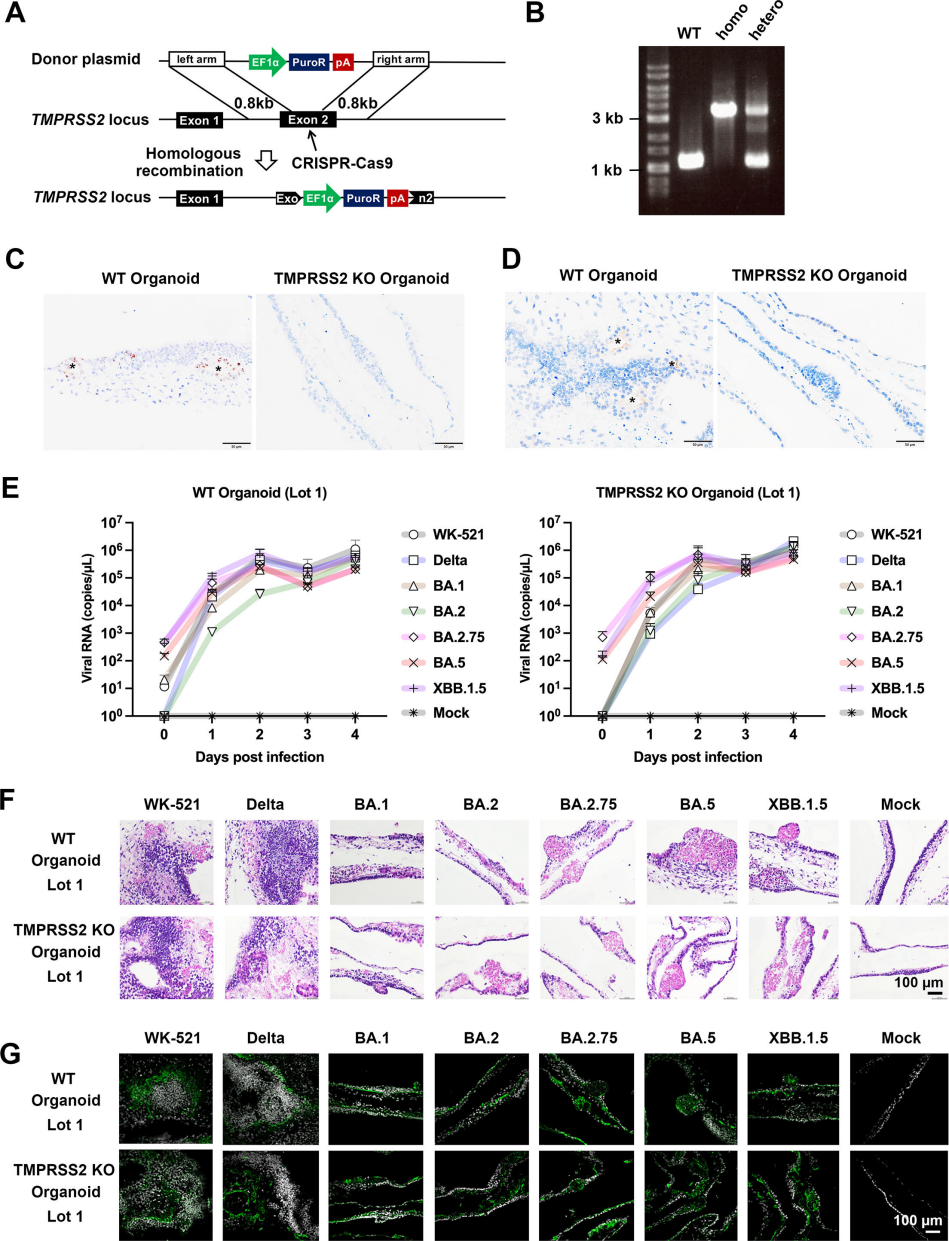
Replication ability in human respiratory organoids. (**A**) A schematic diagram of the targeting strategy for *TMPRSS2* locus. The following donor plasmids were used to target the *TMPRSS2* locus. Donor plasmids: EF1α; elongation factor 1 alpha promoter, PuroR; puromycin resistant protein, pA; polyadenylation sequence. The CRISPR-Cas9 system was utilized to produce TMPRSS2 sequence-specific double-strand breaks. (**B**) Genotyping was performed to examine whether the human iPS cells were correctly targeted. (**C**) RNA *in situ* hybridization. Representative images showing *TMPRSS2* mRNA. Target signals were visualized by 3′,3′ diaminobenzidine (DAB), brown; counterstaining, hematoxylin. Asterisks, glandular structure; bars, 50 µm. (**D**) Immunohistochemistry. Representative images showing TMPRSS2 antigens. Target signals were visualized by DAB, brown; counter-staining, hematoxylin. Asterisks, glandular structure; bars, 50 µm. (**E**) Replication kinetics in WT and TMPRSS2-KO human respiratory organoids (Lot 1) infected with viruses at an MOI of 0.1. Viral RNA copy numbers in the culture supernatants were quantified at 1, 2, 3, and 4 days post-infection (p.i.). Error bars indicate the standard deviations of three biological replicates. Mean values with positive standard deviations are shown. (**F**) H&E staining of uninfected or infected human respiratory organoids. (**G**) Immunofluorescence images of SARS-CoV-2 N protein (green) in uninfected or infected human respiratory organoids. Nuclei were counterstained with DAPI (blue).

Previous studies have demonstrated the importance of TMPRSS2 in SARS-CoV-2 infection in Calu-3 cells and the murine airways (17, 25, 26). Indeed, replication of BA.1 is severely restricted in these systems, at least partly due to its inability to utilize TMPRSS2. However, it should be noted that BA.1 is one of the significant variants that spread globally, replacing the Delta variant. These facts suggest that Calu-3 and the murine airways may only partially reflect actual SARS-CoV-2 infection in humans. Thus, SARS-CoV-2 infection was assessed using induced pluripotent stem cell (iPSC)-derived human bronchoalveolar respiratory organoids. TMPRSS2 gene-knockout (TMPRSS2 KO) respiratory organoids derived from iPSC were also used. Part of the Exon2 region of the TMPRSS2 gene was deleted by homologous recombination with the puromycin-resistant gene using the CRISPR-Cas9 system (Fig. 2A and S2). For a homozygously edited clone, PCR amplification of the gene in this region detected only an approximately 3.5 kb band of the modified gene (Fig. 2B), indicating that recombination at the *TMPRSS2* locus occurred in both alleles. TMPRSS2 KO was also confirmed by transcriptome analysis using total RNAs isolated from both the gene-unmodified (wild type [WT]) and TMPRSS2 KO lung organoids (Fig, S3). In addition, the transcriptome analysis demonstrated some expression level changes in other type II transmembrane serine proteases and cathepsins (Fig. S3). The most notable changes were observed for TMPRSS6. It was increased in TMPRSS2 KO organoids. However, other changes were either at most twofold or expression was deficient in both wild-type and KO organoids. To further confirm TMPRSS2 KO, RNA *in situ* hybridization (ISH) and immunohistochemistry (IHC) were performed using formalin-fixed paraffin-embedded sections of these organoids (Fig. 2C and D) and control cells (Fig. S4). As a result, TMPRSS2 mRNA was detected in WT organoids but not in TMPRSS2 KO respiratory organoids, as strong dot signals in the epithelial cells that had differentiated into glandular structures (Fig. 2C). Furthermore, the TMPRSS2 antigen was detected as linear signals on the luminal side of epithelial cells forming glandular structures in WT organoids but not in TMPRSS2 KO organoids (Fig. 2D).

WT and TMPRSS2 KO organoids were infected with various SARS-CoV-2 strains. Unlike in Calu-3 cells treated with nafamostat, all analyzed SARS-CoV-2 strains, including Omicron BA.1, replicated efficiently in WT and TMPRSS2 KO organoids (Fig. 2E). However, it should be noted that the gene knockout is specific to the target gene, and inhibitors also target related proteases. Therefore, comparing Calu-3 cells treated with nafamostat and TMPRSS2 knockout organoids is challenging. Nevertheless, these data indicate that TMPRSS2 expression is not essential for SARS-CoV-2 infection in human respiratory organoids. Also, histopathologically, there was no apparent difference in the distribution pattern of SARS-CoV-2 antigen between WT organoids and TMPRSS2 KO organoids (Fig. 2F and G). However, when the experiment was repeated three times, marked differences in the replication capacity of SARS-CoV-2 were shown in different organoid lots (Fig. S5). Although strict protocols were followed for organoid differentiation, it was challenging to control the maturity of respiratory organoids at present. However, no significant trend was observed regarding viral growth or antigen detection between WT organoids and TMPRSS2 KO organoids, even when accounting for the lot-to-lot differences (Fig. 2 E through G; Fig. S4 and S6). Although quantitative comparisons in these experiments were therefore difficult, it was reasonable to conclude that TMPRSS2 expression was not essential for SARS-CoV-2 replication in these human respiratory organoids.

### The respective role of TMPRSS2 and cathepsins for SARS-CoV-2 infection in human respiratory organoids

**Fig 3:**
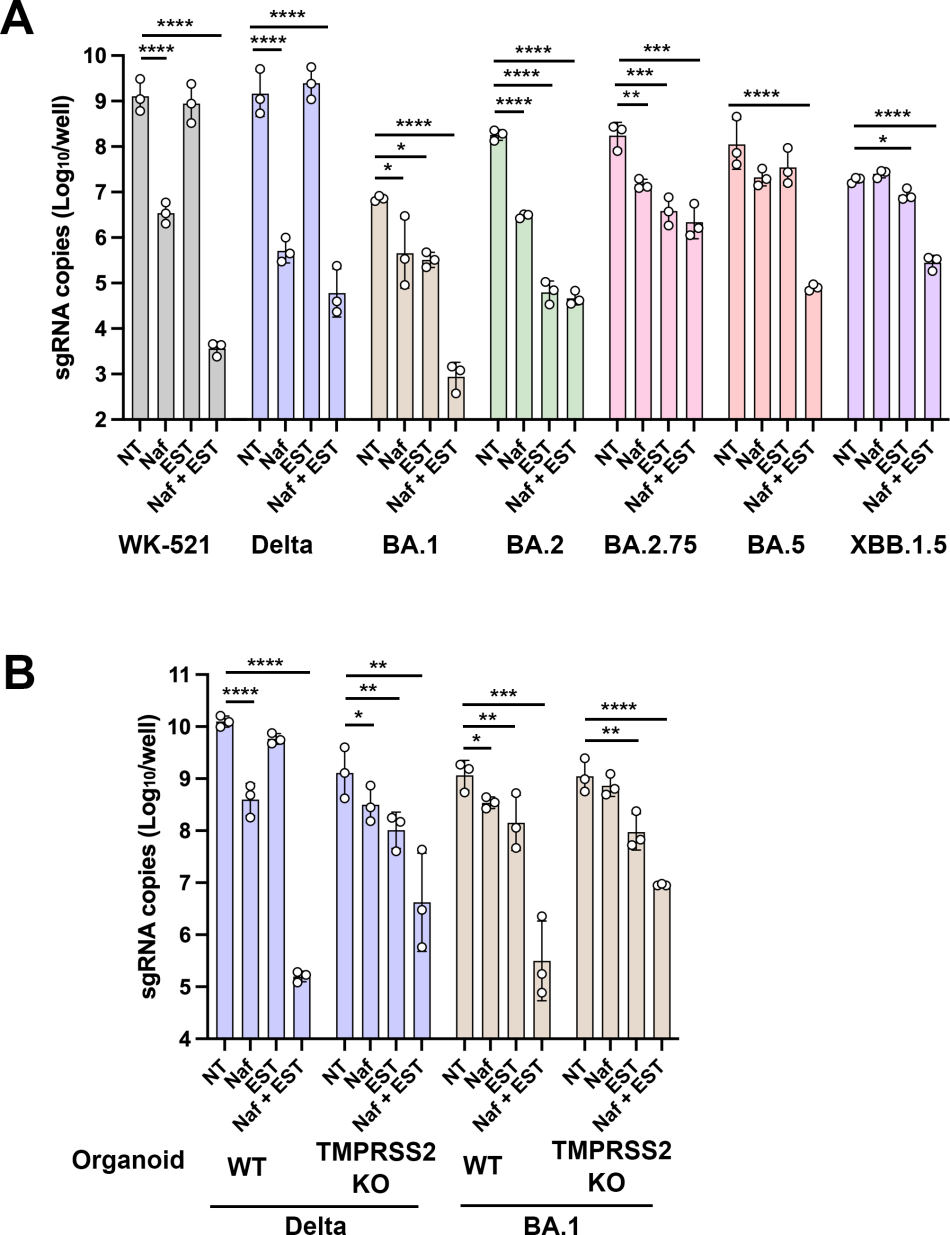
Entry phenotype in human respiratory organoids. (**A**) Wild-type human respiratory organoids were infected with viruses at an MOI of 1.0 in the absence (NT) or presence of either nafamostat (Naf) or EST or both (Naf +EST). At 24 h p.i., viral RNAs in cells were quantified by real-time RT-PCR. Error bars indicate the standard deviations of three biological replicates. Two confirmatory experiments were conducted. Statistical significance was determined using two-way ANOVA. Multiple comparisons between NT and different groups were adjusted with Dunnett’s multiple comparison tests, *P < 0.05, **P < 0.01, ***P < 0.001, ****P < 0.0001. (**B**) Wild-type or TMPRSS2-gene knockout human respiratory organoids were infected with viruses at an MOI of 0.1 in the absence (NT) or presence of either nafamostat (Naf) or EST or both (Naf +EST). At 24 h p.i., viral RNAs in cells were quantified by real-time RT-PCR. Error bars indicate the standard deviations of three biological replicates. Two confirmatory experiments were conducted. Statistical significance was determined using two-way ANOVA. Multiple comparisons between NT and different groups were adjusted with Dunnett’s multiple comparison tests, *P < 0.05, **P < 0.01, ***P < 0.001, ****P < 0.0001.

The primary advantage of using TMPRSS2 KO organoids was the ability to evaluate the role of the TMPRSS2 gene specifically. Unlike in Calu-3 cells and in mouse airways, it was clear that SARS-CoV-2 infection was not strongly dependent on TMPRSS2 in human respiratory organoids. However, a significant disadvantage was the high lot-to-lot variation, which made the quantitative comparison of organoids between WT and TMPRSS2 KO organoids difficult. Therefore, the effects of nafamostat and EST were analyzed to determine the type of protease predominantly utilized by SARS-CoV-2 in WT and TMPRSS2 KO respiratory organoids. In WT respiratory organoids, the WK-521 strain was inhibited by nafamostat by ~2.5 log_10_ (Fig. 3A). The inhibitory effect of EST became evident when it was combined with nafamostat (Fig. 3A). The serine protease(s) primarily responsible for the infection of SARS-CoV-2 in these organoids has not yet been identified (whereas it is TMPRSS2 in Calu-3 [25, 26]), and various serine proteases are shown to activate SARS-CoV-2 (3). These results, therefore, indicate that certain serine protease(s) play a significant role in the WK-521 infection of human respiratory organoids and that the role was more significant than cathepsins (Fig. 3A). Similarly, infection of the Delta variant was also inhibited by nafamostat by ~3.5 log_10_ but poorly by EST (Fig. 3A).

By contrast, the infection of Omicron BA.1 was inhibited by both nafamostat and EST by ~1.0 log_10_ (Fig. 3A). When both drugs were used simultaneously, BA.1 infection was inhibited by ~4 log_10_ (Fig. 3A). Using only one drug resulted in either no or only a small effect on infection with BA.5 and XBB.1.5. However, significant inhibitory effects were confirmed when both drugs were used simultaneously (Fig. 3A). These results suggested that both serine proteases and cathepsins contributed at similar levels in the BA.1, BA.5, and XBB.1.5 infection of respiratory organoids and that the inhibitory effect was only pronounced when both were inhibited. EST inhibited the infection of Omicron BA.2 and BA.2.75 more effectively than nafamostat (Fig. 3A). The addition of nafamostat to EST did not significantly increase the inhibitory effect of EST (Fig. 3A). These data suggested an essential role of cathepsins in these virus infections in human respiratory organoids. Thus, even after recovery of TMPRSS2 availability, the cathepsin-mediated pathway was still important for infection of these Omicron subvariants.

Nafamostat inhibited SARS-CoV-2 infection, although the inhibitory effect varied between SARS-CoV-2 strains (Fig. 3A). However, all SARS-CoV-2 strains replicated efficiently in TMPRSS2 KO organoids (Fig. 2). Do these data suggest that TMPRSS2 plays only a minor role in SARS-CoV-2 infection in respiratory organoids? The effects of nafamostat and EST on SARS-CoV-2 infection were analyzed using TMPRSS2 KO organoids. In TMPRSS2 KO organoids, infection by the Delta variant was inhibited by nafamostat by only ~0.6 log_10_, which was smaller than that observed in WT organoids (~1.5 log_10_). In TMPRSS2 KO organoids, the inhibitory effect of EST was even more significant than that of nafamostat even for the Delta variant. These data suggest a significant role of TMPRSS2 for the Delta variant infection in human respiratory organoids because the greater effect of nafamostat compared to EST, observed for the Delta variant in WT organoids, was not observed in TMPRSS2 KO organoids (Fig. 3B). However, these data also suggested a supplementary contribution of other serine protease(s) because the infection was inhibited by nafamostat by ~0.6 log_10_ even in TMPRSS2 KO organoids (Fig. 3B). The inhibitory effect of nafamostat was attenuated even for the Omicron BA.1 variant in the TMPRSS2 KO organoid (Fig. 3B). Therefore, TMPRSS2 plays a role for the BA.1 infection in human respiratory organoids. However, the contribution of cathepsins was as significant as or more significant than TMPRSS2 in BA.1 infection of these organoids.

## DISCUSSION

Many previous studies have highlighted the importance of TMPRSS2 in SARS-CoV-2 infection both in cultured cells and *in vivo* (3, 17). Indeed, from the Wuhan strain (including the WK521 strain) to the Delta variant, the ability of SARS-CoV-2 to utilize TMPRSS2 has increased gradually, peaking in the Delta variant (29, 30). However, the Omicron variant, which emerged in 2022 and became the dominant global strain, utilizes TMPRSS2 inefficiently (20, 21). These unexpected findings contradict previous studies suggesting that TMPRSS2 is crucial for SARS-CoV-2 infection and spread *in vivo* (17). The spike proteins of Omicron variants are significantly altered in antigenicity, which may have contributed to its dominance (22). However, these changes cannot fully explain why the Omicron variants have become the predominant strains with significantly reduced TMPRSS2 utilization. This study showed that, rather than a single specific protease, various proteases, including TMPRSS2, other serine proteases, and cathepsins, contribute to SARS-CoV-2 infection significantly in the respiratory organoids.

Our study also revealed a gradual recovery in the utilization of TMPRSS2 from the first Omicron BA.1 variant to subsequent Omicron subvariants. These changes were evident in Calu-3 cells, in which SARS-CoV-2 infection is strongly dependent on TMPRSS2, but were challenging to replicate in the respiratory organoids. However, these results suggest that increased TMPRSS2 utilization may confer SARS-CoV-2 an advantage in transmission in human populations, but further studies are needed to understand the role of TMPRSS2 in SARS-CoV-2 transmission. In our respiratory organoids, differentiated populations of both bronchial and alveolar epithelial cells coexisted. Histopathological analysis showed no significant differences in the distribution pattern of viral antigens between WT and TMPRSS2 KO organoids. However, the roles of TMPRSS2 in determining the replication sites of SARS-CoV-2 *in vivo* (upper or lower respiratory tract) and clinical symptoms are areas for future research.

In previous studies with other organoids, Mykytyn et al. (31) showed that camostat, a serine protease inhibitor similar to nafamostat, inhibited infection of the Delta, Omicron BA.1, and Omicron XBB.1.5 variants in respiratory organoids. They concluded that the inhibitory effect of camostat was most likely due to the inhibition of various TTSPs rather than TMPRSS2 alone. Furthermore, analyses using TMPRSS2 KO intestinal organoids showed a dependence on TMPRSS2 for infection of Delta and Omicron BA.1 variant (31). Beumer et al. also showed that TMPRSS2 expression is essential for SARS-CoV-2 infection in intestinal organoids (32). However, they stated that the possible involvement of other TTSPs, inhibited by camostat, such as TMPRSS11A and TMPRSS11D, cannot be ruled out (32, 33). Although the importance of TTSP, including TMPRSS2, is certainly significant, this study has shown the importance of cathepsins comparable to TTSPs in respiratory organoids.

Although the importance of TMPRSS2 in activating viral membrane fusion proteins is undoubted, our study suggests that the contributions of other proteases would also be as significant as TMPRSS2. Given the emergence of various variants with different protease-utilizing capacities, especially Omicron variants, these findings that SARS-CoV-2 infection is not strongly dependent on TMPRSS2 are convincing. Therefore, although broadly effective serine protease inhibitors may be effective against SARS-CoV-2, the effect of TMPRSS2-specific inhibitors may be limited. Nonetheless, developing antiviral drugs targeting protease-mediated activation of viral membrane fusion proteins remains an attractive area of research. To prepare for future pandemics, we aim to understand these activation mechanisms better, improve our understanding of the pathogenesis and transmission of respiratory viruses, and develop new therapeutic strategies.

## MATERIALS AND METHODS

### Cell lines

VeroE6/TMPRSS2 cells were generated in our laboratory previously (7) and deposited in the Japanese Collection of Research Bioresources Cell Bank (the National Institute of Biomedical Innovation, Health and Nutrition) (Cat# JCRB1819). The cells were cultured in Dulbecco’s modified Eagle’s medium (DMEM), high glucose (Cat# D5796, Sigma-Aldrich), containing 10% fetal calf serum (FCS) and antibiotics (100 U/mL penicillin and 0.1 mg/mL streptomycin). The cells were maintained in the presence of 1.0 mg/mL geneticin (G418; Cat# 08973-14, Nacalai Tesque) and were used for analysis without geneticin. The human bronchial epithelial Calu-3 cells were purchased from ATCC (Cat# HTB-55) and cultured with Eagle’s minimum essential media (EMEM, Cat# 051-07615, FUJIFILM Wako Pure Chemical) supplemented with 5% FCS and antibiotics. A subculture lot (NIIDv3 lot) of Calu-3 cells (17) was also used in this study. It has been passaged in DMEM with 10% FCS and antibiotics for dozens of generations in our laboratory and has an improved proliferation ability.

### Human iPS cells

The iPS cells (1383D6) were provided by Dr. Masato Nakagawa, Kyoto University, and maintained on 0.5  µg/cm^2^ recombinant human laminin 511 E8 fragments (iMatrix-511, Cat# 892 012, Nippi) with StemFit AK02N medium (Cat# RCAK02N, Ajinomoto Healthy Supply). The cells were passed every 6 days. For cell passaging, cell colonies were treated with TrypLE Select Enzyme (Cat# 12563029, Thermo Fisher Scientific) for 10 min at 37°C and seeded with StemFit AK02N medium containing 10  µM Y-27632 (Cat# 034–24024, FUJIFILM Wako Pure Chemical).

### Respiratory organoids differentiated from human iPS cells

To start the differentiation, human iPS cell colonies were treated with TrypLE Select Enzyme (Cat# 12563029, Thermo Fisher Scientific) for 10 min at 37°C. After centrifugation, cells were seeded onto Matrigel Growth Factor Reduced Basement Membrane (Cat# 354230, Corning)-coated cell culture plates (2.0 × 10^5^ cells/4 cm^2^) and cultured for 2 days. The differentiation of the respiratory organoids was performed in serum-free differentiation (SFD) medium, composed of DMEM/F12 (3:1) (Cat# 044–29765, FUJIFILM Wako Pure Chemical and Cat# 11320033, Thermo Fisher Scientific) supplemented with N2 (Cat# 141–08941, FUJIFILM Wako Pure Chemical), B-27 Supplement Minus Vitamin A (Cat# 12587001, Thermo Fisher Scientific), ascorbic acid (50 µg/mL, Cat# ST-72132, STEMCELL Technologies), 1 × GlutaMAX (Cat# 35050-061, Thermo Fisher Scientific), 1% monothioglycerol (Cat# 195-15791, FUJIFILM Wako Pure Chemical), 0.05% bovine serum albumin (Cat# 820024, Sigma-Aldrich), and antibiotics (penicillin and streptomycin). During days 0–1 of differentiation, cells were cultured with SFD medium supplemented with 10 µM Y-27632 (Cat# 034–24024, FUJIFILM Wako Pure Chemical) and 100 ng/mL recombinant Activin A (Cat# 338-AC-01M, R&D Systems). During days 1–3 of differentiation, cells were cultured with SFD medium supplemented with 10 µM Y-27632 (Cat# 034-24024, FUJIFILM Wako Pure Chemical), 100 ng/mL recombinant Activin A (Cat# 338-AC-01M, R&D Systems) and 1% fetal bovine serum (FBS). Between days 3 and 5 of differentiation, cells were cultured in SFD medium supplemented with 1.5 µM Dorsomorphin dihydrochloride (Cat# 047-33763, FUJIFILM Wako Pure Chemical) and 10 µM SB431542 (Cat# 037-24293, FUJIFILM Wako Pure Chemical) for 24 h, and then SFD medium supplemented with 10 µM SB431542 and 1 µM IWP2 (Cat# 04-0034, Stemolecule) for another 24 h. During days 5–12 of differentiation, cells were cultured with SFD medium supplemented with 3 µM CHIR99021 (Cat# 034-23103, FUJIFILM Wako Pure Chemical), 10 ng/mL human FGF10 (Cat# AF-100-26, PeproTech), 10 ng/mL human FGF7 (Cat# AF-100-19, PeproTech), 10 ng/mL human BMP4 (Cat# 120–05ET, PeproTech), 20 ng/mL human EGF (Cat# AF-100-15, PeproTech), and all-trans retinoic acid (Cat# R2625 ATRA, Sigma-Aldrich). On day 12 of differentiation, cells were dissociated and embedded in the Matrigel Growth Factor Reduced Basement Membrane to generate organoids. During days 12–20 of the differentiation, organoids were cultured in SFD medium containing 3 µM CHIR99021, 10 ng/mL human FGF10, 10 ng/mL human FGF7, 10 ng/mL human BMP4, and 50 nM ATRA. On day 20 of differentiation, organoids were recovered from the Matrigel, and the resulting suspension of organoids (small free-floating clumps) was seeded onto Matrigel-coated cell culture plates. During days 20–30 of differentiation, organoids were cultured in SFD medium containing 50 nM dexamethasone (Cat# S1322, Selleck Chemicals), 0.1 mM 8-bromo-cAMP (Cat# 1140/50, Tocris), and 0.1 mM IBMX (3-isobutyl-1-methylxanthine) (Cat# 095–03413, FUJIFILM Wako Pure Chemical).

### Human iPS cell electroporation with donor vectors

TMPRSS2 locus was targeted using the donor plasmids and CRISPR/Cas9 RNP. The human iPS cells were electroporated with 4 µg donor plasmids and complex the CRISPR-Cas9 ribonucleoprotein (RNP), 30.5 pmol of Alt-R S. p. Cas9 Nuclease V3 (IDT 108303556), and 30.5 pmol of Hs.Cas9.TMPRSS2.1.AA sgRNA (CGGATGCACCTCGTAGACAG, PAM sequence TGG; IDT 108303552). The electroporation was performed using NEPA21 (NEPAGENE). After the electroporation, the cells were seeded onto LN511-E8-coated dishes, and cultured with the medium containing 10 µM Y-27632. After culturing for 2 days, the medium was replaced with 10 µM puromycin (Cat# 14861-71, INVIVOGEN)-containing medium. The puromycin-containing medium was replaced with the medium 48 h after its addition. After 10 days from the electroporation, colonies were picked up and then seeded onto an LN511-E8-coated 24-well plate. After most of the wells became nearly confluent, genotyping was performed to examine whether the clones were correctly targeted with the following primers: forward (5ʹ-TCACTGCAATCTCTGCCTCC-3ʹ), reverse (5ʹ-AGCCTGAATTAACAGCTGGGA-3ʹ).

### Donor vectors for targeting *TMPRSS2*

To knockin the EF1α-PuroR-pA (elongation factor 1 alpha promoter followed by PuroR protein and polyadenylation sequence) cassette into the *TMPRSS2* locus, a donor template plasmid was constructed. The donor template plasmid was generated by conjugating the following four fragments: two homology arms (0.8 kbp for the 3′ arm and 0.8 kbp for the 5′ arm), an EF1α-PuroR-pA cassette, and linearized backbone plasmids (pENTR donor plasmids). The backbone of donor plasmids was kindly provided by Dr. Akitsu Hotta (Kyoto University) (34).

### Viruses

Viruses were isolated from anonymized clinical specimens (nasopharyngeal/nasal swabs or saliva) collected from individuals diagnosed with COVID-19 as part of the public health diagnostic activities conducted by the National Institute of Infectious Diseases (NIID) (7, 35). VeroE6/TMPRSS2 cells were inoculated with the specimens, cultured in DMEM supplemented with 2% FCS and antibiotics, and observed daily for the appearance of cytopathic effects (CPEs). If necessary, the cells were passaged several times until apparent CPEs were observed. The culture supernatants containing isolated viruses were stored at −80˚C. The nearly full-length genome sequences of isolated viruses were determined as reported previously (35). The data were deposited in the Global Initiative on Sharing All Influenza Data (GISAID) database. The clinical isolates used in this study are shown in Table S1.

### Virus titration

The infectivity titers of stocked viruses were analyzed using VeroE6/TMPRSS2 cells cultured in DMEM containing 2% FCS and antibiotics. Viral infectivity titers were expressed as 50% tissue culture infectious dose (TCID_50_) per milliliter calculated according to the Behrens-Kärber method. All experiments with infectious SARS-CoV-2 were performed under BSL3 conditions.

### SARS-CoV-2 infection

One day before infection, VeroE6/TMPRSS2 cells (4 × 10^4^ cells) and Calu-3 cells (4 × 10^4^ cells) were seeded into a 96-well plate. First, SARS-CoV-2 was inoculated at an MOI of 0.1 and incubated at 37˚C for 1 h. Then, the infected cells were washed, and 100 µL of culture medium was added. The culture supernatant (5 µL) was harvested at the indicated time points, mixed with 45 µL nuclease-free water, and incubated at 99˚C for 5 min. The amount of viral RNA was then quantified by real-time PCR.

### Protease inhibition assay

Calu-3 cells (1 × 10^5^ cells/well) were seeded into a 96-well plate one day before infection. For the assessment of the effect of protease inhibitors [(2S,3S)-trans-Epoxysuccinyl-L-leucylamindo-3-methylbutane ethyl ester (EST) (Cat# 330005, Calbiochem, San Diego, CA, USA) or nafamostat (Cat# N0959, Tokyo Chemical Industry)] on SARS-CoV-2 infection of Calu-3 cells or respiratory organoids, the cell monolayers or respiratory organoids were pre-treated with these inhibitors for 30 min at 37°C. Cells or respiratory organoids were then inoculated with SARS-CoV-2 (an MOI of 0.1 or 1) and incubated with the inhibitors for 1 h at 37°C. The residual virus was removed, and fresh medium containing inhibitors at the indicated concentrations was added to the cells, followed by culture at 37°C for 23 h. Cellular RNA was isolated adding ISOGEN reagent (Cat# 315-02504, Nippon Gene). Then, a real-time PCR assay was performed to ascertain the amount of newly synthesized subgenomic SARS-CoV-2 RNA.

### Detection of SARS-CoV-2 RNAs by real-time RT-qPCR

Real-time RT-qPCR was performed to detect SARS-CoV-2 RNA using the QuantiTect Probe RT–PCR kit (Cat# 204443, Qiagen) with the following primers and probes: SARS-CoV-2_NIID_S_F1 (5ʹ-CAGTCAGCACCTCATGGTGTA-3ʹ), SARS-CoV-2_NIID_S_R3 (5ʹ-AACCAGTGTGTGCCATTTGA-3ʹ), and SARS-CoV-2_NIID_S_P2 (5ʹ-FAM-TGCTCCTGCCATTTGTCATGATGG-BHQ1-3ʹ), and SARS2-LeaderF60 (5ʹ-CGATCTCTTGTAGATCTGTTCTCT-3ʹ), SARS2-N28354R (5ʹ-TCTGAGGGTCCACCAAACGT-3ʹ), and SARS2-N28313Fam (5ʹ-FAM-TCAGCGAAATGCACCCCGCA-TAMRA-3ʹ) for targeting the subgenomic RNA. First, the reaction mixtures were incubated at 50°C for 30 min, followed by incubation at 95°C for 15 min, and thermal cycling, which consisted of 45 cycles of denaturation at 94°C for 15 s, and annealing and extension at 60°C for 60 s. The assay was performed on a LightCycler 480 (Roche, Basel, Switzerland). Data analysis was performed using LightCycler 480 software (version 1.5.1) (Roche). The detection rate (equivalence) of these primer-probe sets for variants, including Omicron, has already been reported (36).

### Detection of cathepsin mRNAs by real-time qRT-PCR

Total RNA was isolated from VeroE6 and Calu-3 (NIIDv3 lot) cells using the Isogen reagent. Real-time PCR was performed to quantify expression of mRNAs encoding glyceraldehyde-3-phosphate dehydrogenase (GAPDH), cathepsin L with the following primers: GAPDH_F (5′-AGAACATCATCCCTGCCTCTACTG-3′), GAPDH_R (5′-CCTCCGACGCCTGCTTCAC-3′), Cathepsin L_F (5’- GAAAGGCTACGTGACTCCTGTG-3′), and Cathepsin L_R (5’- CCAGATTCTGCTCACTCAGTGAG-3′).

### RNA sequencing

Total RNA was isolated from respiratory organoids using ISOGENE. RNA integrity was assessed using a 2100 Bioanalyzer (Agilent Technologies). Library preparation was performed using an Illumina Stranded mRNA Prep Kit (Cat# 20040532, Illumina) according to the manufacturer’s instructions. Sequencing was performed on an Illumina NextSeq2000. The fastq files were generated using bcl2fastq-2.20. Adapter sequences and low-quality bases were trimmed from the raw reads using Cutadapt ver v4.6 (37). For the transcriptome analysis of humans, the trimmed reads were mapped to human reference genome sequences (hg38) using STAR ver 2.7.11a (38) with the GENCODE (release 32, GRCh38.p13) gtf file (39). The raw counts were calculated using htseq-count ver. 2.0.5 (40) with the GENCODE gtf file. Gene expression levels were determined as Transcripts Per Kilobase Million (TPM) values.

### SDS-PAGE and immunoblotting

Stocked viruses were diluted with 4 × sample buffer (Cat# 1610737, Bio-Rad) and boiled for 10 min. The polypeptides were separated by SDS-PAGE and immunoblotted. For protein detection, the following antibodies were used: a mouse anti-SARS-CoV-2 S1 subunit monoclonal antibody (Cat# MAB105403, clone #1035206, R&D systems), a rabbit anti-SARS-CoV-2 S2 subunit polyclonal antibody (Cat# ab272504, Abcam) as the primary antibodies, a horseradish peroxidase (HRP)-conjugated goat anti-mouse IgG (Cat#710–1332, ROCKLAND), and an HRP-conjugated goat anti-rabbit IgG (Cat# 55689, MP Biomedicals) as the secondary antibodies. Chemiluminescence was detected with SuperSignal West Femto Maximum Sensitive HRP Substrate (Cat# 34095, Thermo Fisher Scientific) using Amersham Imager 800 (Cytiva).

### Immunofluorescence staining

For immunofluorescence staining of respiratory organoids, cells were fixed with 4% paraformaldehyde in PBS at 4°C. Respiratory organoids were harvested to prepare paraffin sections (approximately 15 µm). Paraffin was removed using xylene and afterward rehydrated with different percentages of ethanol. Antigen retrieval was performed with 0.1%-tTBS (10×) (pH 7.4) (Cat# 12750–81, Nacalai Tesque). The slides were incubated in Blocking One (Cat# 03953-066, Nacalai Tesque) for 10 min to block non-specific staining at room temperature. The slide was incubated with a rabbit anti-SARS-CoV-2 nucleocapsid protein antibody (Cat# 40588-T62, Sino Biological) overnight at 4°C. On the following day, the stained slides were washed three times with 1 × PBS (Cat# 14249–24, Nacalai Tesque) and incubated with an Alexa Fluor 488-conjugated donkey anti-rabbit IgG (Cat# A21206, Thermo Fisher Scientific) at room temperature for 45  min. The slides were washed afterward three times for 5 min. The sections on the slides were finally washed, and mounted with ProLong Glass Antifade Mountant with NucBlue Stain (Cat# P36985, Thermo Fisher Scientific) and 4′,6′-diamidino-2-phenylindole (DAPI) (Cat# 12593-64; Nacalai Tesque), and analyzed using an inverted laser scanning confocal microscopy system (FV3000, Evident).

### Hematoxylin and eosin staining

Respiratory organoids were fixed with 4% paraformaldehyde (Cat# 163-20145, FUJIFILM Wako Pure Chemical) for 15 min, harvested, and used to prepare paraffin sections. The Applied Medical Research Laboratory performed paraffin-embedded tissue sectioning and histological staining.

### RNA ISH and IHC for TMPRSS2

The human respiratory organoids were fixed in 4% paraformaldehyde for 30 min on ice. HEK293T/17 cells were transfected with TMPRSS2-expressing plasmid, pcDNA3-TMPRSS2 (11), or mock-transfected using Lipofectamine 3000 (Invitrogen). At 2 days post-transfection, TMPRSS2 expressed and non-expressed control cell blocks were prepared using a cell suspension gelatinizing agent (iPGell, GenoStaff, Tokyo, Japan) according to the manufacturer’s protocol and postfixed in 10% buffered formalin for 3 days. All samples were embedded in paraffin and sectioned according to standard protocols. RNA ISH and IHC were conducted using an automated stainer, the Leica Bond RX^m^ (Leica Biosystems, Nussloch, Germany) according to the manufacturer’s protocol. The RNAscope 2.5 HD Detection Reagent (BROWN kit; #322100, Advanced Cell Diagnostics, Newark, CA) and RNAscope 2.5LS probe targeting human TMPRSS2 RNA (Hs-TMPRSS2 for Leica, #470348, Advanced Cell Diagnostics) were used for ISH. Sections stained for TMPRSS2 antigen were processed at pH 9.0 in Bond Epitope Retrieval Solution H2 (AR9640, Leica Biosystems) at 100°C for 20 min. Anti-TMPRSS2 polyclonal rabbit antibody (ab56110, abcam) was used as a primary antibody and the BOND Polymer Refine Detection (DS9800, Leica Biosystems) system was used for IHC using the automated stainer.

### Plasmid construction and syncytium formation assay

The Wuhan-type S protein expression plasmid (VG40589-UT) was purchased from SinoBiological, and the S protein open reading frame was cloned into the pCAGGS vector. The cDNAs of the BA.1, BA.2, BA.2.75, BA.4, BA.5, and XBB.1.5 S genes were reverse-transcribed from purified viral RNAs of strains, hCoV-19/Japan/TY38-873P0/2021, hCoV-19/Japan/TY40-385/2022, hCoV-19/Japan/TY41-716/2022, hCoV-19/Japan/TY41-703/2022, hCoV-19/Japan/TY41-702/2022, and hCoV-19/Japan/23–018/2022, respectively, and cloned into pCAGGS vector. VeroE6/TMPRSS2 cells were transfected with these S protein-expressing plasmids using Lipofectamine LTX (Invitrogen). At 2 days post-transfection, the cells were washed with PBS and fixed with a 10% buffered formalin solution. For syncytia detection, the following antibodies were used: rabbit anti-SARS-CoV-2 S polyclonal antibody (Cat#28867-1-AP, Proteintech) and an Alexa Fluor 549-conjugated goat anti-rabbit IgG (Cat#A-11012, Thermo Fisher Scientific). The nuclear DNA was stained with DAPI. The images were acquired using All-in-One Fluorescence Microscope BZ-X800 (Keyence). The fusion index was calculated as the average number of nuclei per syncytium.

### Statistical analyses

All data are expressed as the mean and standard error of the mean or mean and standard deviation. Statistical analyses were performed using GraphPad Prism 9 software (version 9.4.1). Intergroup comparisons were performed using nonparametric analysis. A *P* value < 0.05 was considered statistically significant. The relevant figure legends indicate the number of independent experiments and technical replicates used. Statistical analyses included ANOVA with multiple correction post-tests.

## Data Availability

Raw data generated from WT and TMPRSS2 KO respiratory organoids were submitted to the Gene Expression Omnibus (GEO) under accession number GSE279753.

## SUPPLEMENTAL MATERIAL

Fig. S1 jvi.01853-24-s0001.tif Cathepsin expression.

Fig. S2 jvi.01853-24-s0002.tif Sequencing analyses in WT and TMPRSS2-KO iPS cells.

Fig. S3 jvi.01853-24-s0003.tif Expression levels of TTSP and cathepsin genes in wild-type (WT) and TMPRSS2 KO respiratory organoids analyzed by RNA-seq.

Fig. S4 jvi.01853-24-s0004.tif Detection of TMPRSS2 expression by RNA *in situ* hybridization and immunohistochemistry.

Fig. S5 jvi.01853-24-s0005.tif Replication kinetics in human respiratory organoids.

Fig. S6 jvi.01853-24-s0006.tif SARS-CoV-2 infection experiment in human respiratory organoids.

Supplemental material jvi.01853-24-s0007.docx Table S1 and legends for Fig. S1 to S6.

ASM does not own the copyrights to Supplemental Material that may be linked to, or accessed through, an article. The authors have granted ASM a non-exclusive, world-wide license to publish the Supplemental Material files. Please contact the corresponding author directly for reuse.
