# Supplementary figures and images for: The respective roles of TMPRSS2 and cathepsins for SARS-CoV-2 infection in human respiratory organoids
Source: J Virol. 2024 Nov 27;99(1):e01853-24. doi: 10.1128/jvi.01853-24 (PMC11784140; doi:10.1128/jvi.01853-24)

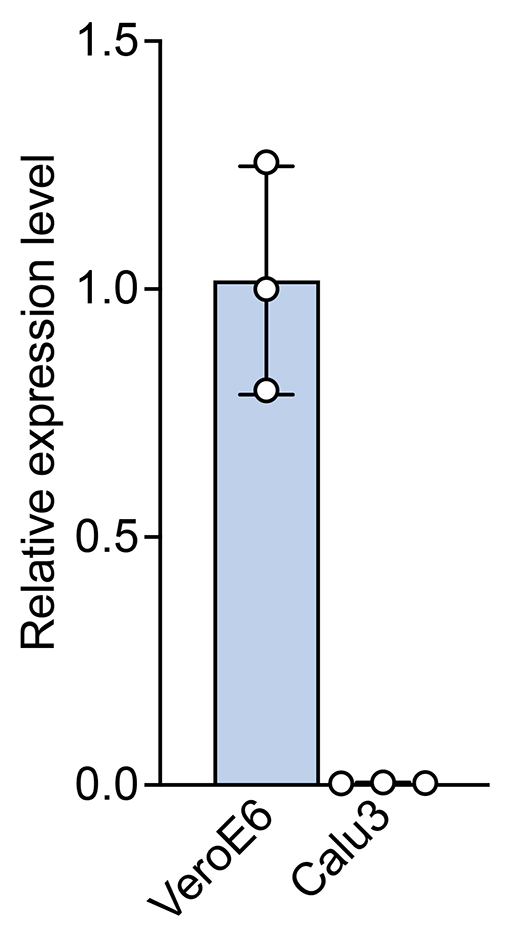

Supplement: Fig. S1 — Cathepsin expression. [file jvi.01853-24-s0001.tif]

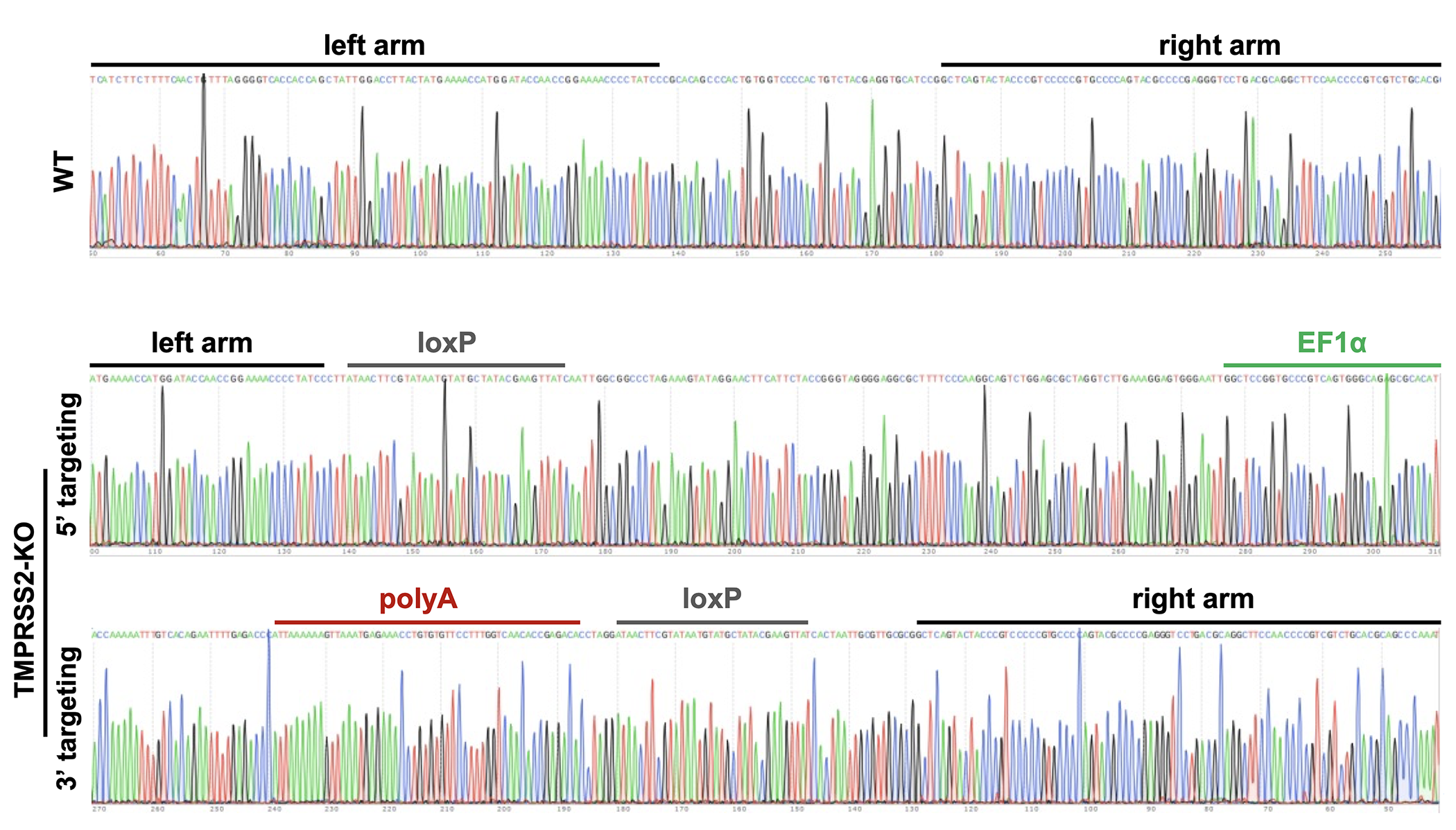

Supplement: Fig. S2 — Sequencing analyses in WT and TMPRSS2-KO iPS cells. [file jvi.01853-24-s0002.tif]

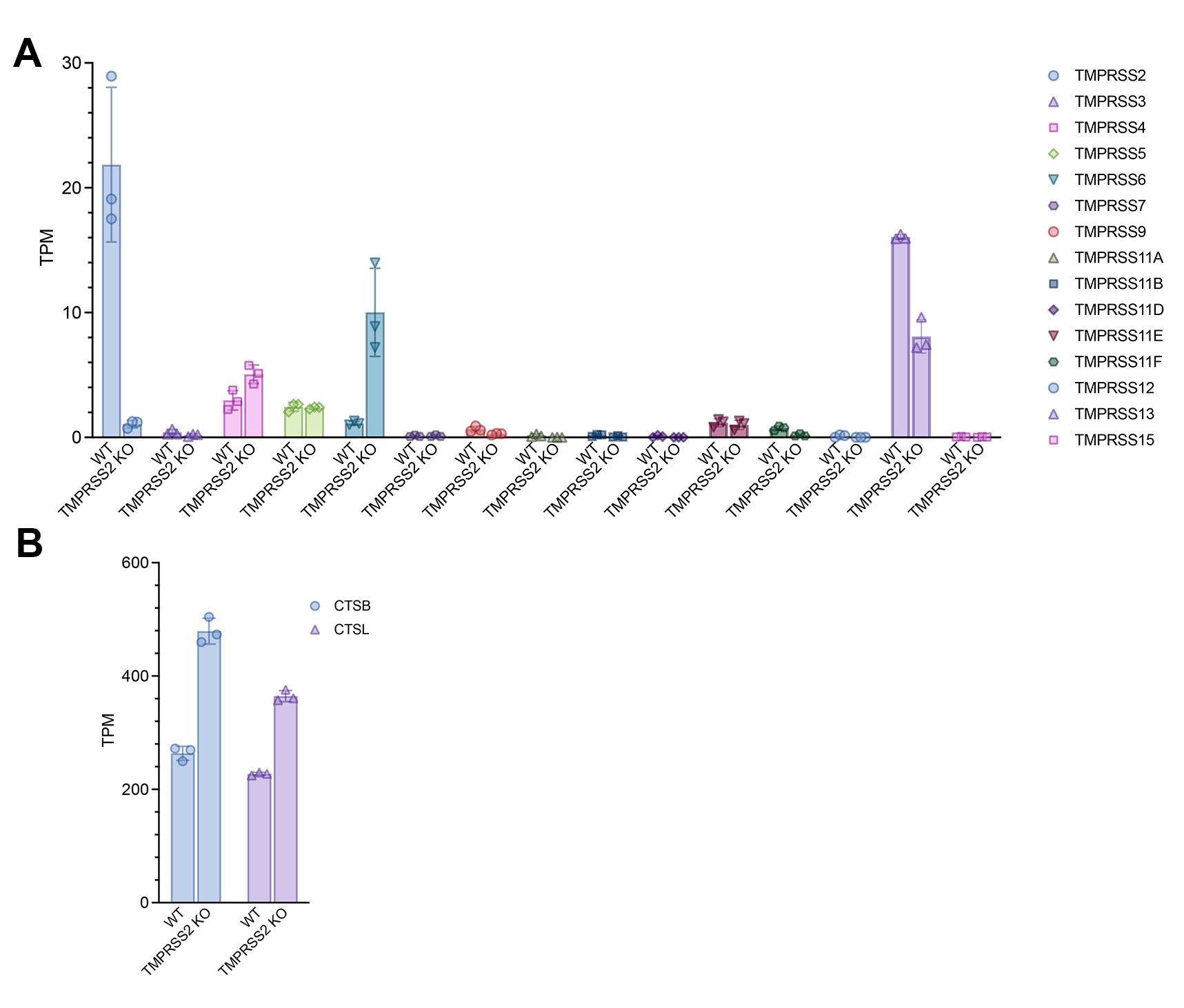

Supplement: Fig. S3 — Expression levels of TTSP and cathepsin genes in wild-type (WT) and TMPRSS2 KO respiratory organoids analyzed by RNA-seq. [file jvi.01853-24-s0003.tif]

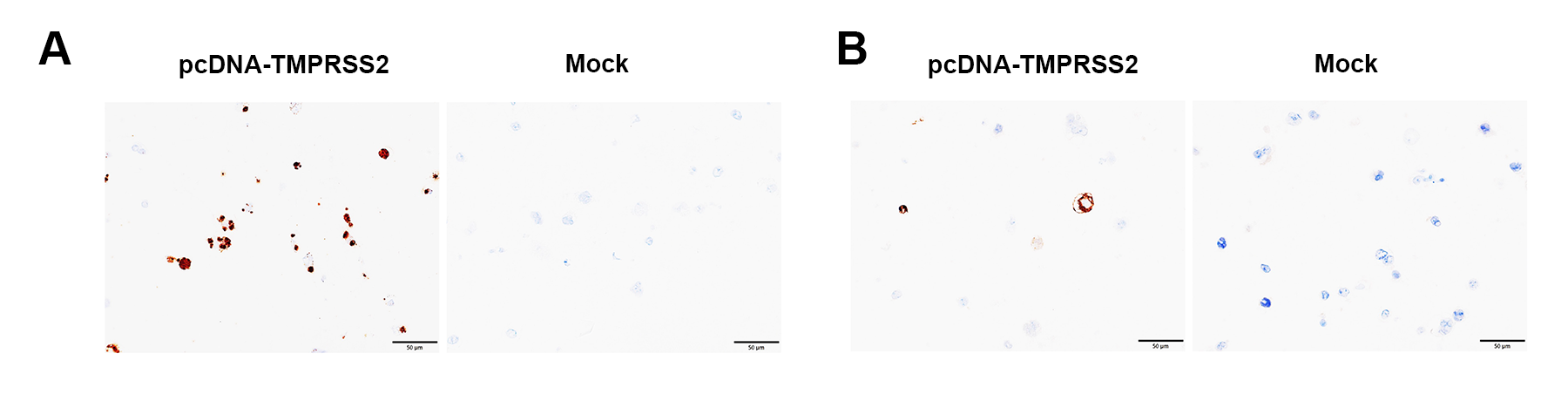

Supplement: Fig. S4 — Detection of TMPRSS2 expression by RNA in situ hybridization and immunohistochemistry. [file jvi.01853-24-s0004.tif]

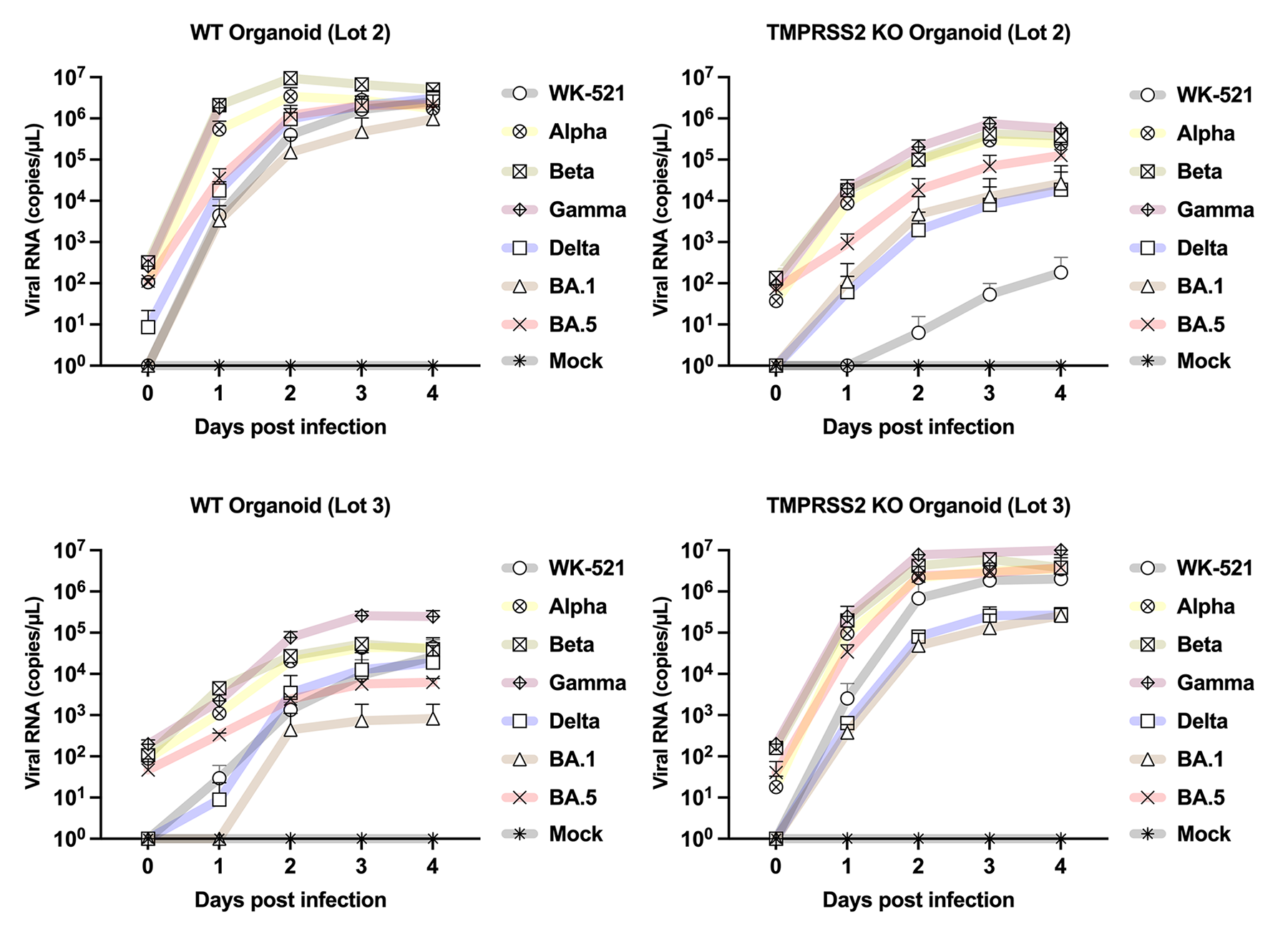

Supplement: Fig. S5 — Replication kinetics in human respiratory organoids. [file jvi.01853-24-s0005.tif]

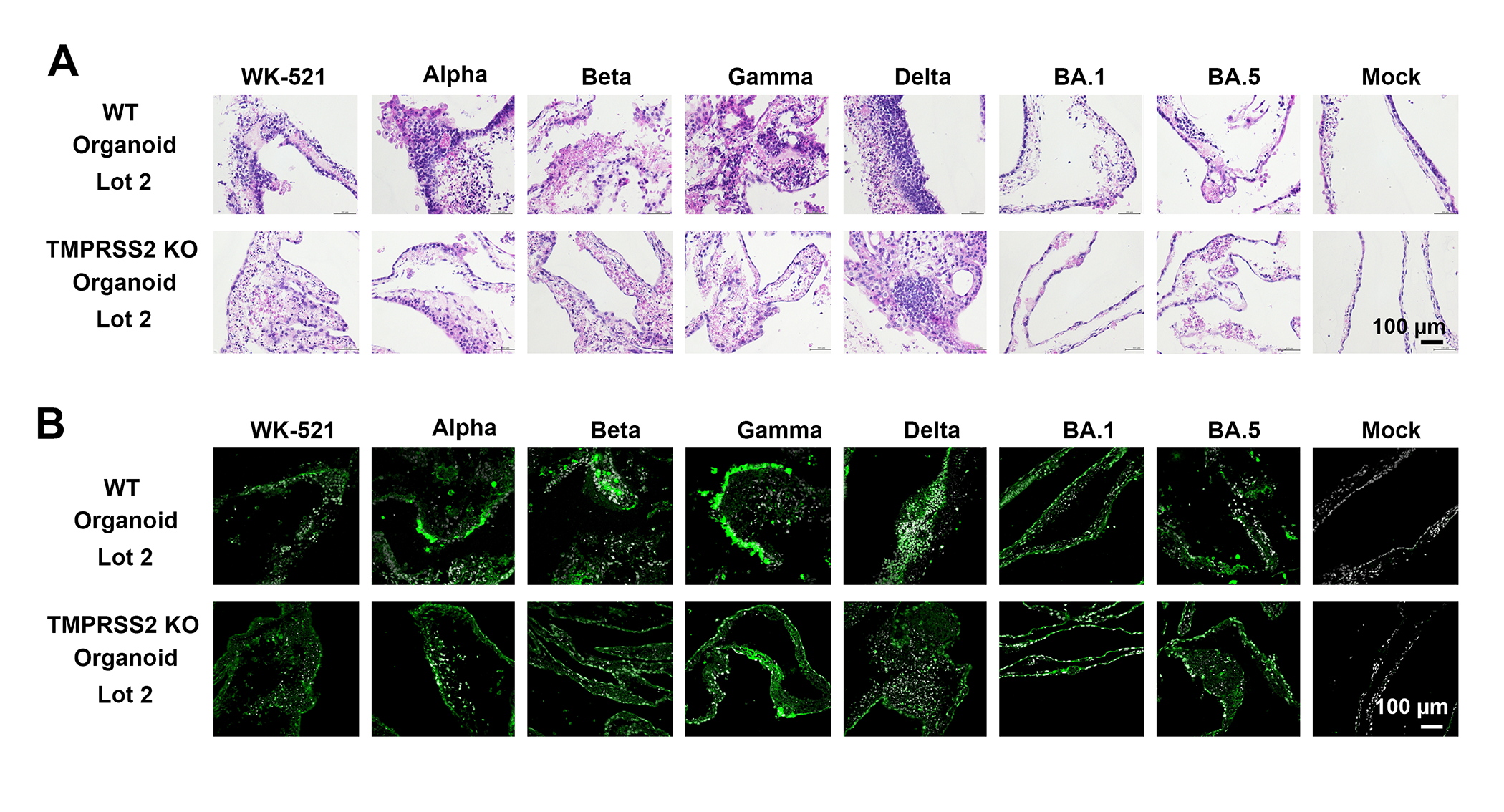

Supplement: Fig. S6 — SARS-CoV-2 infection experiment in human respiratory organoids. [file jvi.01853-24-s0006.tif]
