# Supplementary material for: The respective roles of TMPRSS2 and cathepsins for SARS-CoV-2 infection in human respiratory organoids
Source: J Virol. 2024 Nov 27;99(1):e01853-24. doi: 10.1128/jvi.01853-24 (PMC11784140; doi:10.1128/jvi.01853-24)
Supplement: Supplemental material — Table S1 and legends for Fig. S1 to S6. [file jvi.01853-24-s0007.docx]

**Supplemental Materials**

**Supplemental Table 1. Virus strains used in this study**

| Name | Simplified name | WHO label | Panglin lineage | GISAID accession no. |
| --- | --- | --- | --- | --- |
| hCoV-19/Japan/TY-WK-521/2020 | WK-521 | none | A | EPI_ISL_408667 |
| hCoV-19/Japan/TY8-612-P1/2021 | TY8-612 | Beta | B.1.351 | EPI_ISL_1123289 |
| hCoV-19/Japan/TY7-501/2021 | TY7-501 | Gamma | P.1 | EPI_ISL_833366 |
| hCoV-19/Japan/TY11-927-P1/2021 | TY11-927 | Delta | AY.122 | EPI_ISL_2158617 |
| hCoV-19/Japan/TY38-873P0/2021 | TY38-873 | Omicron | BA.1 | EPI_ISL_7418017 |
| hCoV-19/Japan/TY40-385/2022 | TY40-385 | Omicron | BA.2 | EPI_ISL_9595859 |
| hCoV-19/Japan/TY41-716/2022 | TY41-716 | Omicron | BA.2.75 | EPI_ISL_13969765 |
| hCoV-19/Japan/TY41-703/2022 | TY41-703 | Omicron | BA.4.1 | EPI_ISL_9595828 |
| hCoV-19/Japan/TY41-702/2022 | TY41-702 | Omicron | BA.5 (BE.1) | EPI_ISL_13241867 |
| hCoV-19/Japan/23-018/2022 | 23-018 | Omicron | XBB.1.5.19 | EPI_ISL_16889601 |

**Supplemental Figure Legend**

**Supplemental Fig 1. Cathepsin expression.** *Cathepsin L* mRNA expression in VeroE6 or Calu-3 (NIIDv3 lot) were measured by reverse-transcription quantitative polymerase chain reaction (RT-qPCR), and each sample was normalized relative to *GAPDH* expression. Error bars indicate the standard deviations of triplicate wells. Mean values ± standard deviations are shown. Statistical analysis was performed using student’s *t* test. *P < 0.05.

**Supplemental Fig 2. Sequencing analyses in WT and TMPRSS2-KO iPS cells.** Sequencing analyses were performed to examine whether the TMPRSS2-KO iPS cell clones were correctly targeted. To confirm the DNA sequence, the PCR products were purified and subjected to sequencing analyses.

**Supplemental Fig 3. Expression levels of TTSP and cathepsin genes in wild-type (WT) and TMPRSS2 KO respiratory organoids analyzed by RNA-seq.** The x-axis represents the gene expression levels in Transcripts Per Million (TPM) in WT and TMPRSS2 KO respiratory organoids. The bars indicate the mean TPM values with error bars representing the standard deviation. (A) *TTSPs*. (B) *Cathepsins* (*CTSB* and *CTSL*).

**Supplemental Fig 4. Detection of TMPRSS2 expression by RNA *in situ* hybridization and immunohistochemistry.** Representative images showing *TMPRSS2* mRNA (A) and TMPRSS2 antigens (B). Target signals were visualized by 3′,3′ diaminobenzidine (DAB), brown; counter staining, hematoxylin. Bars, 50 µm.

**Supplemental Fig 5. Replication kinetics in human respiratory organoids.** Replication kinetics in human respiratory organoids (Lot 2 and Lot 3) infected with viruses at an MOI of 0.1. Viral RNA copy numbers in the culture supernatants were quantified at 1, 2, 3, and 4 days post-infection (p.i.). Error bars indicate the standard deviations of triplicate wells. Mean values ± standard deviations are shown.

**Supplemental Fig 6. SARS-CoV-2 infection experiment in human respiratory organoids.** WT and TMPRSS2-KO human respiratory organoids (Lot 2) infected with viruses at an MOI of 0.1. (A) H&E staining of uninfected or infected human respiratory organoids. (B) Immunofluorescence images of SARS-CoV-2 N protein (green) in uninfected or infected human respiratory organoids. Nuclei were counterstained with DAPI (blue).
